# Organizational culture change in U.S. hospitals: a mixed methods longitudinal intervention study

**DOI:** 10.1186/s13012-015-0218-0

**Published:** 2015-03-07

**Authors:** Leslie A Curry, Erika L Linnander, Amanda L Brewster, Henry Ting, Harlan M Krumholz, Elizabeth H Bradley

**Affiliations:** Department of Health Policy and Management, Yale School of Public Health, New Haven, CT USA; Robert Wood Johnson Clinical Scholars Program, Department of Medicine, Yale University School of Medicine, New Haven, CT USA; New York Presbyterian Hospital, New York, NY USA; Center for Outcomes Research and Evaluation, Yale-New Haven Hospital, New Haven, CT USA; Section of Cardiovascular Medicine, Department of Medicine, Yale University School of Medicine, New Haven, CT USA

**Keywords:** Organizational culture, Leadership, Intervention, Hospitals, Quality, Acute myocardial infarction

## Abstract

**Background:**

Improving outcomes for patients with acute myocardial infarction (AMI) is a priority for hospital leadership, clinicians, and policymakers. Evidence suggests links between hospital organizational culture and hospital performance; however, few studies have attempted to shift organizational culture in order to improve performance, fewer have focused on patient outcomes, and none have addressed mortality for patients with AMI. We sought to address this gap through a novel longitudinal intervention study, Leadership Saves Lives (LSL).

**Methods:**

This manuscript describes the methodology of LSL, a 2-year intervention study using a concurrent mixed methods design, guided by open systems theory and the Assess, Innovate, Develop, Engage, Devolve (AIDED) model of diffusion, implemented in 10 U.S. hospitals and their peer hospital networks. The intervention has three primary components: 1) annual convenings of the ten intervention hospitals; 2) semiannual workshops with guiding coalitions at each hospital; and 3) continuous remote support across all intervention hospitals through a web-based platform. Primary outcomes include 1) shifts in key dimensions of hospital organizational culture associated with lower mortality rates for patients with AMI; 2) use of targeted evidence-based practices associated with lower mortality rates for patients with AMI; and 3) in-hospital AMI mortality. Quantitative data include annual surveys of guiding coalition members in the intervention hospitals and peer network hospitals. Qualitative data include in-person, in-depth interviews with all guiding coalition members and selective observations of key interactions in care for patients with AMI, collected at three time points. Data integration will identify patterns and major themes in change processes across all intervention hospitals over time.

**Conclusions:**

LSL is novel in its use of a longitudinal mixed methods approach in a diverse sample of hospitals, its focus on objective outcome measures of mortality, and its examination of changes not only in the intervention hospitals but also in their peer hospital networks over time. This paper adds to the methodological literature for the study of complex interventions to promote hospital organizational culture change.

**Electronic supplementary material:**

The online version of this article (doi:10.1186/s13012-015-0218-0) contains supplementary material, which is available to authorized users.

## Background

Improving outcomes for patients with acute myocardial infarction (AMI) is a priority for hospital leadership, clinicians, and policymakers. Despite an overall reduction in 30-day risk-standardized mortality rates (RSMRs) in recent years, the gap between the highest-performing and lowest-performing hospitals persists [1], with over a twofold difference in RSMRs across hospitals based on data from 2009–2012. Using a positive deviance, mixed methods approach [2,3], we have generated evidence intended to improve hospital performance in AMI care across a range of process and outcome performance indicators. Evidence on improvements in appropriate beta-blocker use [4,5], timeliness of life-saving procedures for people with ST-elevation myocardial infarction [6-8], and, most recently, risk-standardized mortality rates after AMI [6,9] has identified common themes among top-performing hospitals. Prominent in hospitals with top performance were key elements of hospital organizational culture that included clinical engagement and senior management support for quality improvement efforts, effective use of data, strong communication and collaboration across groups, and problem solving that fosters learning and resilience to setbacks.

Previous studies, with some exceptions [10-12], have suggested links between hospital organizational culture and measures of hospital performance [13-22]; however, effective interventions to change organizational culture in order to improve performance have proven elusive and few high-quality studies exist. A 2011 Cochrane review of organizational culture change interventions was unable to draw conclusions about effective approaches for changing culture as no studies met the methodological criteria for inclusion [23]. Prospective studies have largely focused on targeted areas such as emergency departments [24] or specific operating rooms [25] rather than across departments or units, or have shown improvements in staff satisfaction, work attitudes, and safety climate [26,27] but not patient outcomes [28,29]. More comprehensive efforts to improve hospital culture, such as the Robert Wood Johnson Pursuing Perfection program, have illuminated key elements of cultural transformation [20], but have not been well positioned to document improvements in patient outcomes in response to shifts in culture [16]. In summary, despite evidence about the prominence of specific features of organizational culture in top performing hospitals, prospective efforts to evaluate consequential improvements in clinical practices and outcomes through shifts in organizational culture have been disappointing.

We sought to address this gap through a novel longitudinal intervention study, Leadership Saves Lives (LSL), directed at influencing organizational culture in hospitals with the goal of improving evidence-based practices and outcomes for patients hospitalized with AMI. We employed an established theory of organizational culture [30], which has been used widely in the study of healthcare organizations and culture change [31-33]. This theory argues that organizational culture is evolutionary in nature, and is characterized by the shared assumptions, values, and patterns of behavior that enable the hospital to survive in a complex and changing environment [30]. We were also guided by a model of diffusion of innovations (Assess, Innovate, Develop, Engage, Develop (AIDED)) [34] that draws on empirical literature on scale up of public health innovations [35-37] and concepts of molecular biology applied to viral spread [38]. The current study addresses limitations of prior research by using a longitudinal mixed methods approach [39] in a diverse sample of hospitals, including objective outcome measures, and examining changes not only in the intervention hospitals but also in their peer hospital networks over time.

We hypothesize that we will observe 1) positive shifts in key dimensions of hospital organizational culture associated with lower mortality rates for patients with AMI [6,9], 2) increased use of targeted evidence-based practices associated with lower mortality rates for patients with AMI [6,9], and 3) reduced in-hospital AMI mortality. The definition of targets for these outcomes is provided in the ‘Data analysis’ section below. Key dimensions of hospital organizational culture encompass facets that have been previously found to be important in the hospital performance improvement literature and include learning and problem solving, psychological safety, senior leadership support, commitment to the organization, and organizational stress [9,16,40-47] The targeted evidence-based practices in this study derive from our previous work [6] and include monthly meetings with hospital clinicians and emergency medical services (EMS) personnel to review AMI cases, both physician and nurse champions for AMI care, nurses dedicated to the catheterization lab, pharmacist rounding on patients with AMI for hospitals, and creative problem solving. We conceive of these practices as concrete actions and behaviors that are signals for underlying elements of organizational culture. The intervention, described in detail below, is aimed at encouraging and supporting hospitals in implementing and sustaining these approaches.

We are also studying whether and how information spreads across preexisting hospital networks. We hypothesize that we will observe positive but less pronounced changes in facets of organizational culture and use of evidence-based practices in the peer hospital networks of intervention hospitals. Following principles from the AIDED model, the intervention is purposefully designed to catalyze uptake and spread of new ways of working across a network of hospitals over time. Over the 2-year study period, we expect to achieve a deep understanding of both the adoption and the spread of innovations by hospitals in a constantly changing environment, with emphasis on the deepest sort of change —that of organizational culture. The purpose of this paper is to present the theoretical foundation for the study, summarize key elements of the intervention, and describe in detail the study methodology to evaluate the intervention.

### Theoretical foundation

#### Open systems theory

We use an open systems theory [48] framework which suggests that organizations survive within the larger environment by importing information from external sources, converting that information to improve their internal practices, and exporting knowledge to the larger environment. In this import-conversion-export model of organizational behavior, effective management of the organizational boundary is paramount to survival in a changing environment [49]. The role of leadership is to manage this boundary [50] so that the organization can absorb needed external resources, apply these resources to the primary task of the organization, and produce meaningful output for the environment. Productive exchange between the organization and its environment is theorized to promote system performance improvement, the goal of our intervention. While organizations also pursue problem solving and innovation using internal resources, theory suggests that they must also have the capacity to draw from outside in order to successfully adapt to the environment and survive [51]. In this study, we seek to encourage organizational leadership such that the intervention hospitals will promote positive shifts in organizational culture that accelerate learning and improvement, integrate evidence-based practices into the routine work of the organization, and spread these features of organizational culture and practices to other hospitals in their professional networks. The intervention focuses on the potential impact of leadership, broadly conceived, as the core lever for fostering effective and sustained improvements.

#### AIDED model of diffusion of innovations

**Figure 1:**
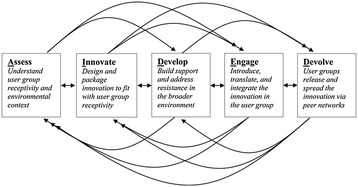
**Components of the AIDED model and relationships among them.**

The AIDED model proposes a parsimonious approach to intervening in a way that promotes further peer-to-peer hospital spread, or diffusion, of the innovation. Although many helpful models of dissemination and diffusion exist [52-55], previous models have not focused on *how* to initiate and sustain spread of innovations. In contrast, the AIDED framework provides practical guidance for how one might plan and implement efforts to spread innovations, and it focuses on achieving such spread within the context of existing resources in the hospital industry. Based on evidence from multiple domestic and global quality collaboratives and campaigns [56,57], as well as biological principles of viral spread [38], the AIDED model posits five interrelated, nonlinear components of what is viewed as a complex adaptive process of innovation adoption and spread (Figure 1). The five components are Assess, Innovate, Develop, Engage, and Devolve (AIDED). *Assess* refers to the process of assessing the environment and the potential user group (e.g., in this study, the intervention hospitals) to understand potential enabling and impeding factors. Assessment may continue periodically throughout the process of spread, supplying needed feedback to the system in order to facilitate adoption and spread. *Innovate* refers to the development of the ideas, practices, and tools that will promote the desired shifts in the hospital. In our case, the innovation is the information about tools to shift organizational culture and processes of care for patients with AMI. *Develop* refers to creating political, regulatory, economic, and technical supports that create an environment conducive to adopting and spreading the innovation. *Engage* encompasses the process from introduction of the innovation to the hospital through fully embedding the innovation in the hospital’s routine processes of care. This component includes three subcomponents: introduce, translate, and integrate. Introduce refers to the process of making a boundary spanner from inside the organization aware of the innovation. Translation refers to the process of reframing the innovation in the language and symbols that are understood within the organization. The last subcomponent, integrate, is the process of embedding the innovation into the routine activity of the organization. *Devolve* refers to the process of the initial adopting organizations passing the innovation to their network for further spread beyond the initial adopting organizations. The application of the AIDED model to multiple aspects of the study design including both the intervention and the evaluation (as described below) represents a novel contribution to the methodological literature on understanding complex interventions [58,59].

## Methods

### Study design

We chose a mixed methods evaluation approach not only to quantify the change in specific outcomes over the intervention period but also to describe in depth the process of organizational change within intervention hospitals. We selected a fully longitudinal convergent design in which qualitative and quantitative data are collected at all time points and analyzed simultaneously [39,60,61]. Longitudinal mixed methods designs are well suited for studying complex changes processes; however, published studies using this method have had notable limitations [62], including lack of attention to temporal information during analysis of qualitative data, limited integration of quantitative and qualitative datasets with respect to time, and lack of plans to manage missing data.

Primary outcomes include 1) shifts in key dimensions of hospital organizational culture associated with lower mortality rates for patients with AMI and 2) use of targeted evidence-based practices associated with lower mortality rates for patients with AMI. A secondary outcome is in-hospital AMI mortality. The quantitative data will measure changes in these defined outcomes. The definition of targets for these outcomes is provided in the ‘Data analysis’ section below. The qualitative data will provide a rich and nuanced description of evolutions in organizational culture as well as insights into whether and how hospitals adopt the recommended strategies and translated them to fit their organizational context. We will integrate the quantitative and qualitative data at the analysis phase in order to develop a comprehensive understanding of the intervention impact and the mechanisms by which the impact may have occurred.

### Intervention guided by the AIDED framework

We used the AIDED model to inform the development of the intervention and the study sample. First, with regard to Assess, we made a substantial investment of time and effort in understanding the broad economic, regulatory, and clinical environment related to care of patients with AMI (developed over a decade of conducting research in this area), as well as determining the initial and ongoing receptivity of both hospital networks and individual hospitals. Second, consistent with Innovate, we sought extensive input during the intervention design phase through three formal meetings with diverse stakeholders and potential end users (American College of Cardiology leaders in Washington, DC; hospital executives and clinicians responsible for care of patients with AMI in both Long Island, NY, and New Haven, CT). Importantly, we are tailoring the structure and content of the intervention to fit each hospital context, with fidelity to core components and content. Third, in terms of Develop, we identified two leverage points in the environment. Value-based purchasing that includes RSMR in the metric has garnered the attention of hospital executives, incentivizing adoption of the evidence-based intervention. In addition, we primed the environment during our engagement with hospital senior management by appealing to the value of organizational prestige by partnering with the Mayo Clinic, a brand known in the healthcare sector for quality and a culture of continuous quality improvement, as well as emphasizing the importance of such changes for success in the new environment of value-based payments. Fourth, we made intentional investment in the Engage component and its three subcomponents (introduce, translate, and integrate) as follows: 1) we created the key role of boundary spanner to be the hospital liaison whose role was to *introduce* new information regarding the intervention to the hospital and continue to be the primary link between the hospital and the intervention team, as well as between the hospital and its peer hospital network; 2) we asked the intervention hospitals to develop a guiding coalition whose role was to *translate* the intervention content within each hospital context; and 3) we focused the intervention on developing a culture that would enable the staff to *integrate* the new practices and approaches to improvement performance into the organization’s DNA, or routine activities. Last, to enable the Devolve component, we designed the study sample to include preexisting peer hospital networks of the intervention hospitals.

**Figure 2:**
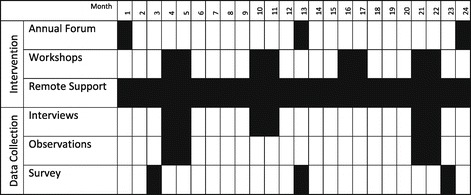
**Intervention and data collection timeline.**

Concretely, the intervention has three primary components: 1) annual forums to convene participants from all ten intervention hospitals; 2) semiannual workshops with guiding coalitions at each hospital; and 3) continuous remote support across all ten hospitals through a web-based platform (Figure 2).

The annual forums at months 0, 12, and 24 bring together four-person teams from each hospital to 1) foster a learning community through providing a safe space for hospitals to share approaches and experiences, 2) promote the translation of the scientific evidence into locally relevant approaches and practices, and 3) support participants as they engage a broader guiding coalition in their hospitals. These in-person annual meetings are 2-days in length and include four participants from each intervention hospital representing diverse roles and perspectives in the care of patients with AMI (a physician and nurse champion, a quality improvement expert, and an executive sponsor). Hospitals send a team that is able to span organizational boundaries, with the authority (formal and informal) and ability to translate new knowledge within their organization; one of the four members is the primary boundary spanner as noted above.

The first annual forum was held in June 2014. At the forum, each hospital was asked to identify a group termed the guiding coalition for the LSL project. The guiding coalition in each hospital is comprised of approximately 15 key staff involved in care of patients with AMI, from senior management to the front line (Table 1). Membership of the guiding coalition is tailored to the existing organizational structure and political context in each intervention hospital. We anticipate that, as the coalition engages across boundaries and identifies priority focal areas for reducing mortality, the membership of the guiding coalition may evolve. Four members of the guiding coalition will participate in annual forums; the full coalition will participate in-hospital workshops and continuous remote support described below, and will complete key informant interviews and annual surveys, also described below. At the conclusion of each annual meeting, participants complete an evaluation form which is used to both assess their immediate perceptions of the quality and utility of the workshop content and format, as well as to inform planning for future meetings.

**Table 1:** **Example membership of guiding coalition**

**Category**	**Example members**
Nursing	Senior executive for nursing
	Nurse champion(s) for AMI care
	Catheterization lab nurse
	Cardiac care unit nurse
	Emergency cepartment nurse
Physician	Senior executive for physicians
	Physician champion(s) for AMI care
	Liaison with EMS
Administration	Senior administrative champion
	Senior executive for quality improvement
	Quality department focal person for AMI
	Data manager
Technicians	Catheterization lab tech
	Emergency department tech
Other	Other front-line workers represented on QI team

The semiannual workshops are designed to strengthen leadership capacity within the guiding coalition and catalyze progress toward improved organizational culture and reduced mortality. To build a culture that supports creative problem solving, workshop content includes teaching and experiential learning on bringing the right perspectives to the table (role definition, group boundaries, working with hierarchy, investing in management capacity), encouraging participants to contribute their individual skills to the common goal (leadership and followership, representational groups, psychological safety), and managing conflict while building accountability within the group. In addition to a focused investment in the culture within the coalition during the workshops, the group is facilitated through a problem solving process [63] to identify root causes of AMI mortality and address those through both integration of external evidence and generation of local solutions [51]. During the first workshop, the guiding coalition prioritizes root causes of AMI mortality and develops strategies to address during the intervention period. Between workshops, they make and measure progress toward their improvement objectives. Subsequent workshops explore implementation challenges and opportunities for continued leadership development. Participants complete an evaluation form at the conclusion of each hospital workshop to report their immediate perceptions of the quality and utility of the workshop, as well as to inform planning for future workshops.

Core leadership concepts are explored by all hospital teams over the course of the four in-hospital workshops, and all teams select priority root causes for focused intervention and improvement. However, workshops are also tailored to each hospital, consistent with the AIDED model’s attention to translation for local context. Tailoring is accomplished by adjusting the timing of modules to meet teams’ most pressing needs, adapting the specific examples and experiential learning exercises used in each module, articulating linkages between these content areas and ongoing work in each hospital, and allowing each hospital to focus on root causes of AMI mortality that are most salient in their environment. A fidelity checklist is completed by facilitators for each round of workshops to ensure consistent delivery of core concepts across sites. Ongoing, systematic evaluation will identify the key components (or ‘active ingredients’) of the workshops with the goal of creating a streamlined and efficient intervention package that is both reproducible and feasible for scale up. Upon completion of the study, products of the intervention including content from each of the workshops and related materials will be made available to hospitals seeking to improve performance in AMI care in their institutions.

We also provide continuous remote support to intervention hospitals through a web-based platform for collaboration called Basecamp [64]. The objectives of the platform are to 1) serve as an accessible, up-to-date repository of LSL materials and references, including both workshop/forum materials and evidence and tools relevant to each of the practices for reducing AMI mortality and 2) enable direct communication across hospital teams and between hospitals and the research team for sharing of successes, barriers, and project updates. Basecamp is a password-protected platform that was selected for ease of use, integration with e-mail communication, and ability to create some project spaces that are open to the full learning community and hospital-specific spaces that are open only to participants from that hospital. On a weekly basis, a member of the research team sends an update to all LSL participants to share tools and new scientific evidence related to AMI mortality, address questions from participants, and promote sharing of experiences across participating hospitals. Between weekly announcements, the site is monitored continuously, and senior members of the research team address participant questions within 1 day of posting. In many cases, questions posed by participants are addressed directly by other participants, promoting peer-to-peer connections and learning. The use of Basecamp will generate data, which can be analyzed to understand who is participating in the online community, and what types of information they are contributing and responding to. Additionally, we include an item on the survey instrument that asks respondents to rate the helpfulness of the Basecamp tool (participant feedback forms are also administered at the end of each workshop and the annual meetings to assess the perceived usefulness of these components of the intervention). Last, we will probe for staff experience using Basecamp during the in-depth interviews. These data, routinely collected and analyzed, will allow us to characterize participant views on and experiences with the remote support component over time.

### Study sample

**Figure 3:**
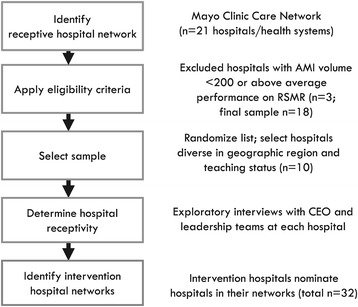
**Hospital sampling process.**

We developed the sample in four stages, using a purposeful random sampling approach [65] (Figure 3). In stage 1, we identified a multihospital network that was receptive to the intervention. We approached the Mayo Clinical Care Network (MCCN), a national membership group of hospitals that complete a due diligence assessment for commitment to patient-centric care, improving quality and safety of clinical practice and delivering value to patients and community. Members access expertise, protocols, and guidelines from the Mayo Clinic. Member organizations are primarily nonprofit, geographically diverse and include academic medical centers, hospital systems, and individual hospitals. We interpreted membership in MCCN as a signal for openness to participation in our intervention. At the time of sample development (January 1, 2014), there were 21 member hospitals/health systems in MCCN.

In stage 2, we selected a purposeful random sample of ten hospitals from within MCCN. Eligibility criteria included 1) 200 or more AMI discharges per year in order to ensure depth of experience in caring for patients with AMI and 2) average or below average national performance on RSMR based on publicly available data from the Centers for Medicare & Medicaid Services Hospital Compare website in January 2014 (reflecting mortality data through June 30, 2012) in order to allow for room to improve. For MCCN members that represented multihospital health systems, we selected the largest or hub hospital from the system for inclusion in the sampling frame. We randomized the list of eligible hospitals (*n* = 18), and beginning from the top, worked in sequence down the randomized list, drawing a sample diverse in teaching status and geographic region (based on U.S. Census categories).

In stage 3, we contacted hospital senior management at selected hospitals to determine organizational receptivity to the intervention. As a marker of receptivity, we asked the executive management from targeted hospitals to commit explicitly in a signed letter of commitment to the following: identify an executive to serve as visible champion for hospital participation; create or select an existing multidisciplinary team of 8–12 staff available for semiannual in-hospital workshops and qualitative data collection (4 days of participation in the intervention and data collection per year); make four senior staff available for travel to annual national convening events; and provide access to documents such as minutes of meetings, and policies and protocols as relevant. After initial consultations with each hospital, two sites declined to participate. One site reported that they had recently started competing initiatives to improve AMI care and thus did not perceive a benefit from participation, and the other hospital was undergoing a transition in executive leadership and was unable to commit to the 2-year intervention. A total of ten hospitals agreed to participate.

In stage 4, we developed the final arm of the sample, the preexisting peer hospital networks of the intervention hospitals. We asked the ten intervention hospitals to identify peer hospitals with whom they regularly share information about caring for patients with AMI. A total of 32 hospitals were nominated (0–7). We obtained contact information for a key staff person responsible for survey completion at each peer hospital, typically a quality improvement director.

### Data collection and measurement

#### Quantitative data

Hospital surveys will be completed at months 0, 12, and 24 of the 24-month intervention period using a secure web-based survey. In the intervention hospitals, all members of the guiding coalition (as defined above) each individually complete a survey. In the peer network hospitals, a single respondent (typically a quality improvement professional) completes a single survey for their hospital.

Measures include dimensions of hospital organizational culture, implementation of evidence-based practices and in-hospital AMI mortality (Table 2). All measures have been used in our previous studies or have been validated by others (survey instrument is included as Additional file 1). To enhance the reliability and validity of data, we conducted cognitive interviews (*n* = 8) [66] to identify cognitive problems with comprehension, recall, or response processes for structured questionnaire items and to revise the instrument accordingly. Data on hospital characteristics will be drawn from the 2010 American Hospital Association Annual Hospital Survey [67]. In addition, for the peer network hospitals, the survey will include a set of items specifically designed to gather information on communication channels as well as the nature of information shared about caring for patients with AMI.

**Table 2:** **Primary outcome measures, independent variables, and covariates**

	**Domain**	**Measures**
Primary outcome measures	Shifts in key dimensions of hospital organizational culture measured by annual survey^b^	*Survey-reported* ^b^ *:*
		• Learning and problem solving
		• Psychological safety
		• Senior leadership support
		• Commitment to the organization
		• Stress/pressure in the system
		*In-depth qualitative interviews*
	Use of five targeted evidence-based practices associated with lower mortality for patients with AMI, measured by annual survey^a^	*Survey-reported:*
		• Monthly meetings between hospital clinicians and EMS to review AMI cases
		• Pharmacist rounds on patients with AMI
		• Nurses specifically assigned to the cardiac catheterization laboratory
		• Both nurse and physician champions
		• Clinicians encouraged to creatively solve problems related to AMI care
		*In-depth qualitative interviews*
	In-hospital AMI mortality	• Total number of deaths of patients with a principal discharge diagnosis of AMI over a 12-month period
Independent variables	Indicator variables for time period	• Pre/post intervention
Covariates	Indicator variable for intervention or network	• Whether hospital is an intervention hospital, or a peer network hospital
	Hospital characteristics	• Multihospital network affiliation
		• Geographic region
		• Teaching status
		• AMI volume

^a^Completed by single respondent per hospital, quality improvement director.

^b^Completed by members of guiding coalition in each hospital (12–15). See survey instrument for items in each dimension of culture.

#### Qualitative data

Prior to each hospital workshop, we complete in-depth interviews [65] with approximately 15 key informants (i.e., the guiding coalition members and nominated others). We conduct additional interviews until we reach theoretical saturation at each site. Interviews are approximately 45 min in length and are audiotaped and transcribed by professional transcriptionists.

Data collection is conducted using a standard interview guide (Additional file 2) to explore the participants’ experience with implementing evidence-based practices in the context of LSL. Questions explore sources of resistance to implementing change, how resistance was managed, and approaches to tailoring the change packet and related tools. To assess how actively members of the guiding coalition are engaging in key actions expected, we collect data to characterize actions undertaken by the guiding coalition including their internal and external advocacy (or lack thereof) for LSL with hospital staff and staff of peer network hospitals, translating information gained at workshops to material that can be used in their hospital, and leading or supporting LSL strategy implementation efforts. We also gather data to assess the changing impact of people in leadership roles in the hospitals, particularly the degree to which they are supportive or hindering of adoption of recommended practices and features of organizational culture. Finally, we conduct selective observations (Additional file 3) of key interactions in care for patients with AMI (e.g., patient rounds, cardiovascular service line department meetings) as well as relevant meetings and other onsite activities. Field notes are transcribed and will inform analyses of the qualitative data. Last, site visit teams participate in a debriefing session promptly upon return from visits; reflections and observations are synthesized in written form and will also inform analyses.

### Data analysis

#### Quantitative data

We will use standard frequency analysis to describe changes in hospital organizational culture, changes in evidence-based practices, and in-hospital AMI mortality over the 2-year study time period and three waves of data collection (at 1, 12, and 24 months) for the overall sample as well as for intervention and peer network hospitals separately. We have created subscales of organizational culture using factor analysis, and will use standard frequency analysis to measure changes on each subscale over time. Our primary endpoints are improvements in the number of recommended practices implemented and improvement in key dimensions of organizational culture. The target for the endpoint of number of recommended practices is the adoption of at least two new practices in each intervention hospital by the end of the study period. The target for the endpoint of improvement organizational culture is achievement in each intervention hospital of a mean score of 2 or lower (on a scale of one to five) in at least two more dimensions of culture that at baseline (a score of 2 or lower reflects an organizational culture that supports high performance in care of patients with AMI). In terms of the secondary outcome, in-hospital AMI mortality, we will measure change in crude mortality (the total number of deaths of patients with a principal discharge diagnosis of AMI) over three 6-month periods (October 1, 2013 to March 31, 2014 for the first survey wave, updated annually for each subsequent wave).

In this longitudinal analysis, each hospital will serve as its own control. We will summarize using descriptive statistics the trend in mortality across hospitals over the study period and assess using unadjusted analyses if this trend differs significantly for intervention versus peer hospitals. In addition, using a repeated measures design, we will conduct multivariable analyses to estimate the associations between time and each of the dependent variables, adjusting for hospital participation status (i.e., as an intervention hospital versus peer network hospital), and relevant hospital characteristics (e.g., geographic region, teaching status, AMI volume). We will explore whether the time effect estimated by the multivariable analysis differs significantly for the intervention versus the peer network hospitals by testing interaction effects. All models will be estimated independently by two data analysts for quality assurance using SAS version 9.3 (Cary, NC). Information from the peer network hospital surveys will be analyzed to determine whether and how aspects of the intervention may have spread to the peer hospitals over time.

#### Qualitative data

Analyses will be carried out by a six-member multidisciplinary team using coding techniques for qualitative data [65] and the constant comparative method [65,68,69]. Coding will occur in iterative steps, in which codes are refined during analysis of transcripts from successive interviews. Team members will independently code all transcripts and then discuss in several joint sessions, and will assign codes to observations by a negotiated, group process. We will conduct systematic analysis of these data in order to characterize whether and how the intervention facilitated or constrained implementation of recommended practices and how key aspects of organizational culture may have changed. Data will be entered into ATLAS.ti (Berlin, Germany) to facilitate analysis. As recommended by experts [65,69], we will search for disconfirming evidence, interview multiple respondents at each hospital for triangulation, and maintain a detailed audit trail to document analytic decisions. We will generate thematic output [68], which will describe recurrent and unifying concepts across the dataset, as well as inform the generation of hypotheses for exploration in future studies.

#### Integration of quantitative and qualitative data

We will integrate the quantitative and qualitative data on evidence-based practices and dimensions of organizational culture at the completion of all data collection and analysis, an approach referred to as ‘merging.’ We will examine the full set of quantitative and qualitative data in order to identify patterns and major themes in change processes across all intervention hospitals over time, with particular attention to temporal information [62]. The qualitative data will also be used to triangulate and extend the quantitative data, and we will also systematically search for and address disconfirming data across the datasets to enhance credibility of our findings [70]. Having both quantitative and qualitative data will allow us to develop a more comprehensive understanding than is possible with one form of data alone, a core principle of mixed methods designs [60,71,72].

#### Limitations

Despite its novelty, our study has several limitations to be considered. First, the primarily qualitative approach and relatively small, purposeful sample limits the generalizability of findings; however, we will enhance transferability of findings [65,73] through ‘thick description’ [70] of longitudinal data characterizing experiences of ten highly diverse hospitals. Second, we are not randomizing hospitals to intervention and nonintervention groups as would be done in a randomized controlled trial (RCT); however, RCTs are not well suited when the unit of randomization is complex (such as hospitals) or when evaluating a ‘real world’ intervention that cannot satisfy the control conditions required for RCTs [74], as is the case with our study. Third, due to the 18-month lag in availability of 30-day RSMR data, we are instead assessing in-hospital AMI mortality rates. While in-hospital rates are not risk-standardized, we do not expect that the risk profile of a given hospital would change substantially over the 2-year period, and we are not comparing mortality across hospitals. Fourth, social desirability response bias [75], or Hawthorn effects [76], may occur. To minimize these effects, we will have multiple on-site observation points, interview multiple staff members in each hospital, use scripted probes to elicit details that would be difficult to misrepresent, instruct respondents to share both positive and negative experiences, and triangulate between interview and survey data [3,65].

## Discussion

Quality of care for patients with AMI has improved substantially in recent years due to important investments by clinicians and policymakers; however, notable variation in survival rates across U.S. hospitals persists. Extensive literature from within healthcare [13-22], as well as from broader business sectors [77,78], suggests that organizational culture shapes the performance of institutions in important ways; nevertheless, little is known about how to create and sustain an organizational culture that fosters excellence in healthcare. This longitudinal intervention and mixed methods evaluation draws upon a deep interdisciplinary literature, as well as the AIDED model of spread of innovations, representing a novel contribution to the methodological literature on developing and testing complex interventions in health care. We seek to advance our understanding of whether and how hospital organizational culture can be changed in order to improve performance on a clinical outcome that matters, and where we know we can do better: mortality of patients with AMI.

## Conclusions

LSL is novel in its use of a longitudinal mixed methods approach in a diverse sample of hospitals, its focus on objective outcome measures of mortality, and its examination of changes not only in the intervention hospitals but also in their peer hospital networks over time. This paper adds to the methodological literature for the study of complex interventions to promote hospital organizational culture change.

## Additional files

Additional file 1:
**Primary LSL baseline survey.**
Additional file 2:
**LSL interview guide.**
Additional file 3:
**LSL selective observation guide.**

## Abbreviations

AIDED: Assess, Innovate, Develop, Engage, Devolve
AMI: Acute myocardial infarction
EMS: Emergency medical services
LSL: Leadership Saves Lives
MCCN: Mayo Clinical Care Network
RCT: Randomized controlled trial
RSMR: Risk-standardized mortality rate
