# Supplementary material for: Organizational culture change in U.S. hospitals: a mixed methods longitudinal intervention study
Source: Implement Sci. 2015 Mar 7;10:29. doi: 10.1186/s13012-015-0218-0 (PMC4356105; doi:10.1186/s13012-015-0218-0)
Supplement: Additional file 1: — Primary LSL baseline survey. [file 13012_2015_218_MOESM1_ESM.pdf]

Primary LSL Baseline Survey

Acute Myocardial Infarction (AMI) Patient Care Survey

Directions: We are conducting a survey to better understand how your hospital cares for patients with AMI. If your hospital is part of a multi-hospital system, please answer on behalf of your hospital only. All information you provide is completely confidential, electronically secure, and only accessible by researchers at the Yale School of Public Health. We will not disclose the source of information or report or publish data in a way that identifies you or your organization. Patient-level data are not collected. This study was approved by the Yale Human Investigations Committee (HIC: 1406014118). The survey will take approximately 20 minutes to complete. We appreciate your time.

Q1 1. What best describes the department you work in?

- ❍ Cardiac Catheterization Laboratory (1)
- ❍ Quality Improvement/Management (2)
- ❍ Corporate Suite (3)
- ❍ Emergency Department (4)
- ❍ Step Down or Telemetry Unit (5)
- ❍ Cardiac Intensive Care Unit (ICU) (6)
- ❍ Other (7) ____________________

Q2 2. What best describes your job function?

- ❍ Physician (1)
- ❍ Physician Assistant (2)
- ❍ Advanced Practice Nurse (3)
- ❍ Nurse (4)
- ❍ Technician (5)
- ❍ Analyst (6)
- ❍ Senior Management/Leadership Team (7)
- ❍ Other (8) ____________________
- ❍

Q3 3. Is your hospital a member of a multi-hospital system?

- ❍ Yes (1)
- ❍ No (2)

Q4 4. What multi-hospital system does your hospital belong to?

Hospital Protocols and Processes for AMI Care

If you are uncertain about the protocols or processes inquired about in this section, please feel free to collaborate with your colleagues to determine the proper response.

Q5 5. Does your hospital track its risk-standardized 30-day mortality rate for patients with AMI?

- ❍ Yes (1)
- ❍ No (2)

Q6 6. Does your hospital track its in-hospital mortality rate for patients with AMI?

- ❍ Yes (1)
- ❍ No (2)

Q7 7. Does your hospital have a quality improvement team(s) that works on reducing 30-day mortality for patients with AMI?

- ❍ Yes (1)
- ❍ No (2)

Q8 8. Is reducing AMI mortality a hospital-wide objective?

- ❍ Yes (1)
- ❍ No (2)

Q9 9. Does your hospital have one or more physician champions (someone who advocates and supports) focused on reducing 30-day mortality in patients with AMI?

❍ Yes (1)
❍ No (2)

Q10 10. Does your hospital have one or more nurse champions (someone who advocates and supports) focused on reducing 30-day mortality in patients with AMI?

❍ Yes (1)
❍ No (2)

Q11 11. Does your hospital have any of the following providers on site twenty four hours a day, seven days a week (24/7):

	Select an answer for parts a-c	
	YES (1)	NO (2)
a. Interventional cardiologist (1)	❍	❍
b. Non-interventional cardiologist (2)	❍	❍
c. Cardiology Fellow (4)	❍	❍

Q12 12. Does your hospital have a designated person or group to review the deaths of patients with AMI (i.e., on an individual case level) that occurred:

	Select an answer for a and b	
	YES (1)	NO (2)
a. During hospitalization? (1)	❍	❍
b. Within 30 days of admission (even if outside of your hospital)? (2)	❍	❍

Q13 13. Do clinicians (e.g., physicians, physician assistants, nurses) from your hospital meet or liaise with emergency medical system (EMS) providers to review the care of patients with AMI?

❍ Yes, about monthly (1)
❍ Yes, about quarterly (2)
❍ Yes, about annually (3)
❍ Yes, other (4) ____________________
❍ No (5)

Q14 14. Does your hospital employ an EMS coordinator whose primary role is to help communicate with EMS agencies?

- ❍ Yes (1)
- ❍ No (2)

Q15 15. Do EMS providers participate in hospital quality improvement efforts regarding AMI?

- ❍ Yes (1)
- ❍ No (2)

Q16 16. Does your hospital use root cause analysis or a similar method to understand problems in AMI care?

- ❍ Yes (1)
- ❍ No (2)

Q17 17. Is your hospital part of a regional effort or consortium of hospitals to improve AMI care?

- ❍ Yes (1)
- ❍ No (2)

Q18 18. Are nurses in any of your critical care areas cross-trained to cover in the cardiac catheterization laboratory?

- ❍ Yes (1)
- ❍ No (2)

Q19 19. Do pharmacists round on all patients with AMI?

- ❍ Yes (1)
- ❍ No (2)

Please answer the following questions about patients admitted to your hospital between October 1, 2013 and March 31, 2014 with a principal discharge diagnosis of AMI. See below for a working definition of AMI.

Q20 20. What was the total number of patients admitted between October 1, 2013 and March 31, 2014 who had a principal discharge diagnosis of AMI? Please see below for a working definition of AMI.

Q21 21. How many of these patients had a discharge disposition of deceased (regardless of cause)?

Q22 22. Did you use ICD9 or ICD10 coding definitions to define this patient population?

- ❍ ICD9 (1)
- ❍ ICD10 (2)

AMI as defined based on ICD 10 Code I21: ST elevation (STEMI) and non-ST elevation (NSTEMI) myocardial infarction. The measure includes beneficiaries with a principal discharge diagnosis of AMI (International Classification of Diseases, Tenth Revision, Clinical Modification, code I21). This excludes beneficiaries with a principal discharge diagnosis of old myocardial infarction (I25.2), postmyocardial infarction syndrome (I24.1), subsequent myocardial infarction (I22.-), and patients seen in emergency or observation units only (not admitted). AMI as defined based on ICD 9 Code 410: Acute myocardial infarction. The measure includes beneficiaries with a principal discharge diagnosis of AMI (International Classification of Diseases, Ninth Revision, Clinical Modification, codes 410.00, 410.01, 410.10, 410.11, 410.20, 410.21, 410.30, 410.31, 410.40, 410.41, 410.50, 410.51, 410.60, 410.61, 410.70, 410.71, 410.80, 410.81, 410.90, and 410.91). This excludes beneficiaries with a principal diagnosis of AMI in subsequent episode of care (410.02, 410.12, 410.22, 410.32, 410.42, 410.52, 410.62, 410.72, 410.82 410.92) and patients seen in emergency or observation units only (not admitted).

Organizational Dynamics

In responding to the following statements, please draw on your own experiences in your current role working with clinical staff and administration to improve AMI care.

Q23 23. The senior management at your hospital has set reducing 30-day mortality after AMI as a priority.

- ❍ Strongly Agree (1)
- ❍ Agree (2)
- ❍ Neutral (3)
- ❍ Disagree (4)
- ❍ Strongly Disagree (5)

Q24 24. Opinion leaders at your hospital have indicated that your current practices for patients with AMI can be improved. (Opinion leaders are defined as staff members who are widely respected and considered influential in the hospital.)

- ❍ Strongly Agree (1)
- ❍ Agree (2)
- ❍ Neutral (3)
- ❍ Disagree (4)
- ❍ Strongly Disagree (5)

Q25 25. Opinion leaders at your hospital have encouraged changes in practices to improve AMI care. (Opinion leaders are defined as staff members who are widely respected and considered influential in the hospital.)

- ❍ Strongly Agree (1)
- ❍ Agree (2)
- ❍ Neutral (3)
- ❍ Disagree (4)
- ❍ Strongly Disagree (5)

Q26 26. In this hospital, the necessary financial resources for personnel and equipment are provided for the care of patients with AMI.

- ❍ Strongly Agree (1)
- ❍ Agree (2)
- ❍ Neutral (3)
- ❍ Disagree (4)
- ❍ Strongly Disagree (5)

Q27 27. I would be very happy to spend the rest of my career at this hospital.

- ❍ Strongly Agree (1)
- ❍ Agree (2)
- ❍ Neutral (3)
- ❍ Disagree (4)
- ❍ Strongly Disagree (5)

Q28 28. I enjoy discussing my hospital with people outside of it.

- ❍ Strongly Agree (1)
- ❍ Agree (2)
- ❍ Neutral (3)
- ❍ Disagree (4)
- ❍ Strongly Disagree (5)

Q29 29. I feel as if this hospital's problems are my own problems.
- ❍ Strongly Agree (1)
- ❍ Agree (2)
- ❍ Neutral (3)
- ❍ Disagree (4)
- ❍ Strongly Disagree (5)

Q30 30. I think I could easily become as attached to another hospital as I am to this one.
- ❍ Strongly Agree (1)
- ❍ Agree (2)
- ❍ Neutral (3)
- ❍ Disagree (4)
- ❍ Strongly Disagree (5)

Q31 31. I do not feel like 'part of the family' at this hospital.
- ❍ Strongly Agree (1)
- ❍ Agree (2)
- ❍ Neutral (3)
- ❍ Disagree (4)
- ❍ Strongly Disagree (5)

Q32 32. I do not feel 'emotionally attached' to this hospital.
- ❍ Strongly Agree (1)
- ❍ Agree (2)
- ❍ Neutral (3)
- ❍ Disagree (4)
- ❍ Strongly Disagree (5)

Q33 33. This hospital has a great deal of personal meaning to me.
- ❍ Strongly Agree (1)
- ❍ Agree (2)
- ❍ Neutral (3)
- ❍ Disagree (4)
- ❍ Strongly Disagree (5)

Q34 34. I do not feel a strong sense of belonging to my hospital.
- ❍ Strongly Agree (1)
- ❍ Agree (2)
- ❍ Neutral (3)
- ❍ Disagree (4)
- ❍ Strongly Disagree (5)

Q35 35. The clinicians who care for patients with AMI hold each other accountable for high quality care.
- ❍ Strongly Agree (1)
- ❍ Agree (2)
- ❍ Neutral (3)
- ❍ Disagree (4)
- ❍ Strongly Disagree (5)

Q36 36. Our hospital has frequent interactions with outside organizations (e.g., other hospitals and professional associations) to acquire new knowledge on how to improve AMI care.
- ❍ Strongly Agree (1)
- ❍ Agree (2)
- ❍ Neutral (3)
- ❍ Disagree (4)
- ❍ Strongly Disagree (5)

Q37 37. There is good coordination among the different clinical units involved with the care of patients with AMI.
- ❍ Strongly Agree (1)
- ❍ Agree (2)
- ❍ Neutral (3)
- ❍ Disagree (4)
- ❍ Strongly Disagree (5)

Q38 38. In this work environment, people value new ideas.
- ❍ Always (1)
- ❍ Usually (2)
- ❍ Sometimes (3)
- ❍ Rarely (4)
- ❍ Never (5)

Q39 39. In this work environment, people are interested in better ways of doing things.
- ❍ Always (1)
- ❍ Usually (2)
- ❍ Sometimes (3)
- ❍ Rarely (4)
- ❍ Never (5)

Q40 40. In this work environment, people often resist new approaches.
- ❍ Always (1)
- ❍ Usually (2)
- ❍ Sometimes (3)
- ❍ Rarely (4)
- ❍ Never (5)

Q41 41. If you make a mistake in this work environment, it is held against you.
- ❍ Always (1)
- ❍ Usually (2)
- ❍ Sometimes (3)
- ❍ Rarely (4)
- ❍ Never (5)

Q42 42. People in this work environment are able to bring up problems and tough issues.
- ❍ Always (1)
- ❍ Usually (2)
- ❍ Sometimes (3)
- ❍ Rarely (4)
- ❍ Never (5)

Q43 43. In this work environment, someone would deliberately act to undermine my efforts.
- ❍ Always (1)
- ❍ Usually (2)
- ❍ Sometimes (3)
- ❍ Rarely (4)
- ❍ Never (5)

Q44 44. It is difficult to ask others in this work environment for help.
- ❍ Always (1)
- ❍ Usually (2)
- ❍ Sometimes (3)
- ❍ Rarely (4)
- ❍ Never (5)

Q45 45. In this work environment, people’s unique skills and attributes are valued and utilized.
- ❍ Always (1)
- ❍ Usually (2)
- ❍ Sometimes (3)
- ❍ Rarely (4)
- ❍ Never (5)

Q46 46. In this work environment, people caring for patients with AMI are overly stressed.
- ❍ Always (1)
- ❍ Usually (2)
- ❍ Sometimes (3)
- ❍ Rarely (4)
- ❍ Never (5)

Q47 47. Despite the workload, people in this work environment find time to review how the work is going.
- ❍ Always (1)
- ❍ Usually (2)
- ❍ Sometimes (3)
- ❍ Rarely (4)
- ❍ Never (5)

Q48 48. In this work environment, the time pressure gets in the way of doing a good job.
- ❍ Always (1)
- ❍ Usually (2)
- ❍ Sometimes (3)
- ❍ Rarely (4)
- ❍ Never (5)

Q49 49. In this work environment, people are too busy to invest time in improvement.
- ❍ Always (1)
- ❍ Usually (2)
- ❍ Sometimes (3)
- ❍ Rarely (4)
- ❍ Never (5)

Q50 50. There is simply no time for reflection in this work environment.
- ❍ Always (1)
- ❍ Usually (2)
- ❍ Sometimes (3)
- ❍ Rarely (4)
- ❍ Never (5)

Q51 51. Clinicians (e.g., physicians, physician assistants, nurses) in this work environment are encouraged to creatively solve problems related to AMI care processes.
- ❍ Always (1)
- ❍ Usually (2)
- ❍ Sometimes (3)
- ❍ Rarely (4)
- ❍ Never (5)

Q52 52. In this work environment, we rely on data to guide our improvement processes.
- ❍ Always (1)
- ❍ Usually (2)
- ❍ Sometimes (3)
- ❍ Rarely (4)
- ❍ Never (5)

Q53 53. Clinicians in this work environment frequently seek new information that leads us to make important changes.
- ❍ Always (1)
- ❍ Usually (2)
- ❍ Sometimes (3)
- ❍ Rarely (4)
- ❍ Never (5)

Q54 54. In this work environment, someone makes sure that we stop to reflect on the team's work process.

- ❍ Always (1)
- ❍ Usually (2)
- ❍ Sometimes (3)
- ❍ Rarely (4)
- ❍ Never (5)

Q55 55. People in this work environment speak up to challenge assumptions.

- ❍ Always (1)
- ❍ Usually (2)
- ❍ Sometimes (3)
- ❍ Rarely (4)
- ❍ Never (5)
