# Supplementary material for: Organizational culture change in U.S. hospitals: a mixed methods longitudinal intervention study
Source: Implement Sci. 2015 Mar 7;10:29. doi: 10.1186/s13012-015-0218-0 (PMC4356105; doi:10.1186/s13012-015-0218-0)
Supplement: Additional file 2: — LSL interview guide. [file 13012_2015_218_MOESM2_ESM.pdf]

LSL Interview Guide
9_14_14

ESTABLISH TRUST
LISTEN INTENTLY
BE CURIOUS AND (APPROPRIATELY) INQUISITIVE
PURSUE UNANTICIPATED DIRECTIONS

We are interested in understanding your hospital's experience with implementing LSL. Are you familiar with LSL (if not, briefly describe). We would like to hear about your experiences including successes, struggles and surprises.

We would like your permission to record this interview for two reasons. First, this lets us listen carefully to you rather than taking notes. Second, it means we will accurately capture our conversation for analysis. All information will be kept strictly confidential and no identifying information about you or your organization is included on the transcript. Digital files with audio recorded material will be deleted as soon as the interview transcripts have been reviewed for accuracy. If at any point you would like me to turn off the recorder, please let me know. You are free to decline to participate, to end our interview at any time for any reason, or to choose to skip any question. Choosing not to participate will involve not affect your relationship with the hospital.

1. Please describe your background and your role at this hospital. We realize you are just getting started, but please also describe your role on the LSL guiding coalition.

   Alternate for participants not on the LSL guiding coalition: Please describe what you do at this hospital and your role relative to the LSL project.

   Provide comfortable, non-threatening a way into the interview; begin to establish a relationship; locate the person in the organization from his/her own perspective; and gain a sense of their role in both the larger process of AMI care and the LSL team

   If respondent appears to be unfamiliar with LSL, shift framing to efforts to improve quality of AMI care or efforts to reduce AMI mortality

2. Your hospital has been engaged in LSL for just a short while. Can you talk a little bit about how it has been going so far?

   Elicit descriptions of LSL coalition's work on processes for AMI care. Give the interviewer the opportunity to explore a broad range of factors that the interviewee considers relevant to AMI patient care in this setting.

3. What aspects of the work have been going smoothly?

   Identify elements of the environment that are facilitating efforts (structural, procedure, relational).

4. We are interested in hearing more about setbacks or sources of resistance to the team's efforts. Can you describe any bumps in the road so far? Any challenges that you think might lie ahead?

   Provide safe space to describe possible sources of tension or friction in the process of change. Uncover principles, strategies, and practices for managing change.

5. Have there been surprises so far? Can you talk a little bit about those?

6. Is there anything we haven't talked about that you would like to add?

Guidance for interviewers: At each step along the interview, the interviewer should **follow the story and ask questions to help us understand the story.** For instance, when asked about a the LSL initiative, the respondent might then say "We put a team together" to which the interviewer might ask "Who put the team together? Who was on it? Why were these people included? What were your aspirations and concerns when you found out you were on it?"

General probes:

- Can you tell me more about that?
- What did you mean when you said "___________"?
- Listen for description of things that got better or worse, and probe why

Content probes: USE JUDICIOUSLY

1. Role
    - What is your formal title?
    - To whom do you report (get title and level)?
    - How long have you worked here? How long have you worked in this job?
    - What are your formal responsibilities?
    - What is your role on the LSL team?

2. Strategies/AMI care processes
    - monthly meetings between hospital clinicians and emergency medical services (EMS) to review AMI cases;
    - assigning nurses specifically to catheterization laboratory (to reinforce and sustain teamwork within the lab);
    - having a pharmacist round on all patients with AMI (unless the hospital always has a cardiologist on site);
    - having both nurse and physician champions for quality in AMI care; and
    - fostering an organizational environment in which clinicians are encouraged to creatively solve problems.

3. QI efforts and culture
    - Data feedback
    - Learning culture
    - Readiness to change
    - Coordination across disciplines
    - Openness to innovation
    - Psychological safety
    - Time to reflect
    - Problem solving
    - Accountability
    - Other education/collaboratives/QI teams

4. Components of LSL intervention
    - LSL workshops (in hospital)
    - LSL online community
    - LSL annual meetings (national – 10 hospitals)
