# Supplementary material for: Organizational culture change in U.S. hospitals: a mixed methods longitudinal intervention study
Source: Implement Sci. 2015 Mar 7;10:29. doi: 10.1186/s13012-015-0218-0 (PMC4356105; doi:10.1186/s13012-015-0218-0)
Supplement: Additional file 3: — LSL selective observation guide. [file 13012_2015_218_MOESM3_ESM.pdf]

LSL Selective Observation Guide
7_9_14

ESTABLISH TRUST
LISTEN INTENTLY
BE CURIOUS AND HUMBLE
OBSERVE IN THE BACKGROUND

Selective observations (1 hour each)	Year 1		Year 2		Total hours
Rounding on patients with AMI	✓			✓	2
Cardiovascular service line department meeting	✓			✓	2
Hospital management committee meeting	✓			✓	2
Guided walk-around in CCU or other nursing unit	✓			✓	2
Guided walk-around in ED	✓			✓	2

Observations of rounding and meetings

We are interested in understanding how you provide care for patients with AMI at this hospital. We would like to observe your team at work to learn how you do things here.

Physical Environment/ Context	• Where is the interaction taking place?<br>• What is the space like?<br>o Cluttered/Neat? Quiet/Loud? Energetic/Relaxed? Bright/Dim? Other?<br>o Who sits or stands where relative to others?
Actors/ Participants	• Who is doing what?<br>• What are the relationships between participants?<br>o Who interacts with whom?<br>o Who empowers or silences whom?<br>o How is power and authority exercised?<br>• Who is missing?
Timing	• What is the frequency and duration of the meeting/rounds you are observing?<br>• Is this a new or established processes?
Informal factors	• What non-verbal cues do you observe?<br>• What visual cues to you observe?<br>• What symbols or symbolic acts do you observe?

Structured observations of patient care units

We are interested in understanding how your hospital provides care for patients with AMI. To help us understand your experiences, we would like to see and learn about what it's like on your patient care units.

Guiding questions (only if needed):

- Can you show me the physical layout of the unit?
- Tell me a bit about the staffing on this unit?
- Where do people go for breaks?
- Where do staff have meetings or discussions with/about patients?
- How do staff communicate with each other and with other departments?
- What is it like for patients and families arriving and departing from the unit?
- Is there anything unique about this unit that we should know about?

Physical Environment/ Context	• What is the space like?<br>• Cluttered/Neat? Quiet/Loud? Energetic/Relaxed? Bright/Dim? Other?<br>• What artifacts of culture do you observe?
Actors/ Participants	• Who is doing what?<br>• What are the relationships between participants?<br>o Who interacts with whom?<br>o Who empowers or silences whom?<br>o How is power and authority exercised?<br>• Who is missing?
Timing	• What is the timing of your visit (morning/evening, busy/slow, shift change, tied to other cyclical patterns?
Informal factors	• What non-verbal cues do you observe?<br>• What visual cues to you observe?<br>• What symbols or symbolic acts do you observe?

Debrief notes

Your observations will be used to inform interpretation of the interview data. At the end of each set of observations, please create a debrief note (approx 2 pages per hour of observation) that summarizes the experience with an emphasis on organizational culture.
